# A double-blind, randomized, placebo-controlled trial of a computer-based Interpretation Bias Training for youth with severe irritability: a study protocol

**DOI:** 10.1186/s13063-018-2960-5

**Published:** 2018-11-14

**Authors:** Simone P. Haller, Joel Stoddard, Caroline MacGillivray, Kelsey Stiles, Gretchen Perhamus, Ian S. Penton-Voak, Yair Bar-Haim, Marcus R. Munafò, Melissa A. Brotman

**Affiliations:** 1 Section on Mood Dysregulation and Neuroscience, Emotion and Development Branch, National Institute of Mental Health, 9000 Rockville Pike, Building 15K, Bethesda, MD 20892 USA; 2 Department of Psychiatry, University of Colorado School of Medicine, Aurora, USA; 3 Lewis Katz School of Medicine, Temple University, Philadelphia, USA; 4 Department of Psychology, University of California, Los Angeles, USA; 5 School of Experimental Psychology, University of Bristol, Bristol, UK; 6 School of Psychological Sciences, Tel Aviv University, Tel Aviv, Israel; 7 Sagol School of Neuroscience, Tel Aviv University, Tel Aviv, Israel; 8 MRC Integrative Epidemiology Unit, University of Bristol, Bristol, UK; 9 UK Centre for Tobacco and Alcohol Studies, University of Bristol, Bristol, UK

**Keywords:** Irritability, Children and adolescence, Cognitive training, Interpretation bias, Randomized controlled trial, RCT, DMDD, Face-emotion

## Abstract

**Background:**

Severe, chronic, and impairing irritability is a common presenting clinical problem in youth. Indeed, it was recently operationalized as disruptive mood dysregulation disorder (DMDD) in the DSM-5. However, to date, there are no evidence-based treatments that were specifically developed for DMDD. The current randomized controlled trial assesses the efficacy of a computer-based cognitive training intervention (Interpretation Bias Training; IBT) in youth with DMDD. IBT aims to reduce irritability by altering judgments of ambiguous face-emotions through computerized feedback. IBT is based on previous findings that youth with irritability-related psychopathology rate ambiguous faces as more hostile and fear producing.

**Methods/design:**

This is a double-blind, randomized controlled trial of IBT in 40 youth with DMDD. Participants will be randomized to receive four IBT sessions (Active vs. Sham training) over 4 days. Active IBT provides computerized feedback to change ambiguous face-emotion interpretations towards happy interpretations. Face-emotion judgments are performed pre and post training, and for 2 weeks following training. Blinded clinicians will conduct weekly clinical ratings. Primary outcome measures assess changes in irritability using the clinician-rated Affective Reactivity Index (ARI) and Clinical Global Impressions-Improvement (CGI-I) scale for DMDD, as well as parent and child reports of irritability using the ARI. Secondary outcome measures include clinician ratings of depression, anxiety, and overall impairment. In addition, parent and child self-report measures of depression, anxiety, anger, social status, and aggression will be collected.

**Discussion:**

The study described in this protocol will perform the first RCT testing the efficacy of IBT in reducing irritability in youth with DMDD. Developing non-pharmacological treatment options for youth suffering from severe, chronic irritability is important to potentially augment existing treatments.

**Trial registration:**

ClinicalTrials.gov, ID: NCT02531893. Registered on 25 August 2015.

**Electronic supplementary material:**

The online version of this article (10.1186/s13063-018-2960-5) contains supplementary material, which is available to authorized users.

## Background

Irritability, defined as an increased proneness to anger and outbursts relative to peers [1, 2], is highly prevalent [3–7] and impairing in youth [8]. Irritability in youth predicts the development of depression and anxiety [3, 4, 7], suicidality [9, 10], and is linked to overall reduced academic and socioeconomic functioning [11]. Given the functional impairment and association with negative outcomes, chronic irritability is a significant public health concern [3–5, 11, 12].

Despite this pressing clinical and public health need, there is remarkably little treatment research on pathological irritablity [1, 2, 13, 14]. Recently, disruptive mood dysregulation disorder (DMDD), with a core feature of chronic irritability, has been introduced into the *Diagnostic and Statistical Manual of Mental Disorders 5* (DSM-5 [15]). DMDD provides a diagnostic category for trials of treatments targeting severe and impairing irritability in youth that may be readily translated into clinical practice. Currently, non-pharmacological treatment strategies for DMDD are investigational, with no evidence-based cognitive trainings to treat the irritability characteristic of DMDD.

### Rational for treatment intervention

Clinically, irritability is characterized by a low threshold for angry, reactively aggressive responses to provocations [14]. One of the most established findings in youth with irritability-related psychopathology is that they are more likely to judge ambiguous social stimuli as hostile [14, 16]. Compared to healthy youth, chronically irritable youth preferentially attend towards threatening (i.e., angry) faces [17], and rate ambiguous faces as fear-inducing [18] or threatening (i.e., interpretation bias; [19]). A substantial body of work has shown that these biased interpretations of ambiguous social stimuli elicit and potentiate angry, aggressive responses in youth [16, 20]. Hence, biases in interpreting social stimuli may serve to maintain irritability [14, 19] and provide a potential target for novel interventions.

One promising set of novel interventions relies on computer-based technology to change biases in information processing through repeated exposure to “corrective” feedback (e.g., [21]). This approach has shown promise in the treatment of anxiety (e.g., [22]). In the study described in this protocol, we will test whether such an approach can be used to shift biased interpretations of face-emotions in DMDD and thereby reduce irritability.

The Interpretation Bias Training (IBT) task [23] is a method to both quantify and train face-emotion interpretation bias. In this task, participants are presented with face-emotions morphed from very happy to very angry across a continuum of 15 stimuli (see Fig. 2 for example stimuli). Participants are instructed to make quick, binary happy-angry judgments for each face morph that appears on the screen. Morphs are presented in random order. Data indicate that both healthy participants and psychiatric patients categorize each morph reliably [23]. The morph in the continuum at which each participant’s judgment switches from happy to angry has been termed “balance point” (BP) and represents a measure of interpretation bias. For instance, participants who rate only a few faces along the happy-angry continuum as happy before switching their judgment to predominantly angry responses would have a low BP, i.e., an increased bias towards perceiving ambiguous faces as angry. This IBT task can also be used to change face-emotion interpretations by providing trial-by-trial, computer-based feedback to shift the participant’s BP along the continuum of face morphs.

In other phenotypes in youth, such as disruptive behavioral problems, preliminary data suggest that shifting participants’ BP may reduce anger and aggression [24]. In a randomized controlled trial (RCT) of 46 adolescents who were in residential treatment because of aggressive behavior and high-risk of criminal behavior, Penton-Voak et al. [24] reported that four sessions of Active compared to Sham training on the IBT task shifted participants’ BP toward happy and away from angry judgments. Further, Active training was associated with a significant reduction in both self-rated anger and aggressive behavior rated by blinded residential treatment staff.

In a recent preliminary study, Stoddard et al. [19] extended this work to youth with DMDD. In a series of pilot experiments, Stoddard and colleagues demonstrated a difference in BP between DMDD (*n* = 63) and healthy youth (*n* = 26), with DMDD youth exhibiting a bias towards angry judgments. Additionally, Stoddard and colleagues tested the feasibility of training irritable youth in an open pilot trial. In 14 DMDD youth, four sessions of Active training resulted in a shift in BP, which persisted for 2 weeks post training. Clinical data were consistent with an improvement in parent-reported irritability over the course of the open pilot trial. While promising, these data are preliminary and do not test efficacy, supporting a test of IBT on irritability in DMDD in a RCT. Thus, the objective of the current study, described in this protocol, is to assess the efficacy of IBT in reducing irritability in youth with DMDD in a double-blind, randomized, placebo-controlled design. The IBT RCT compares Active training, which encourages a shift in face-emotion interpretations toward happy judgments, to Sham training, where feedback does not encourage change in a person’s pre-training face-emotion interpretations.

## Methods/design

### Design

**Fig. 1:**
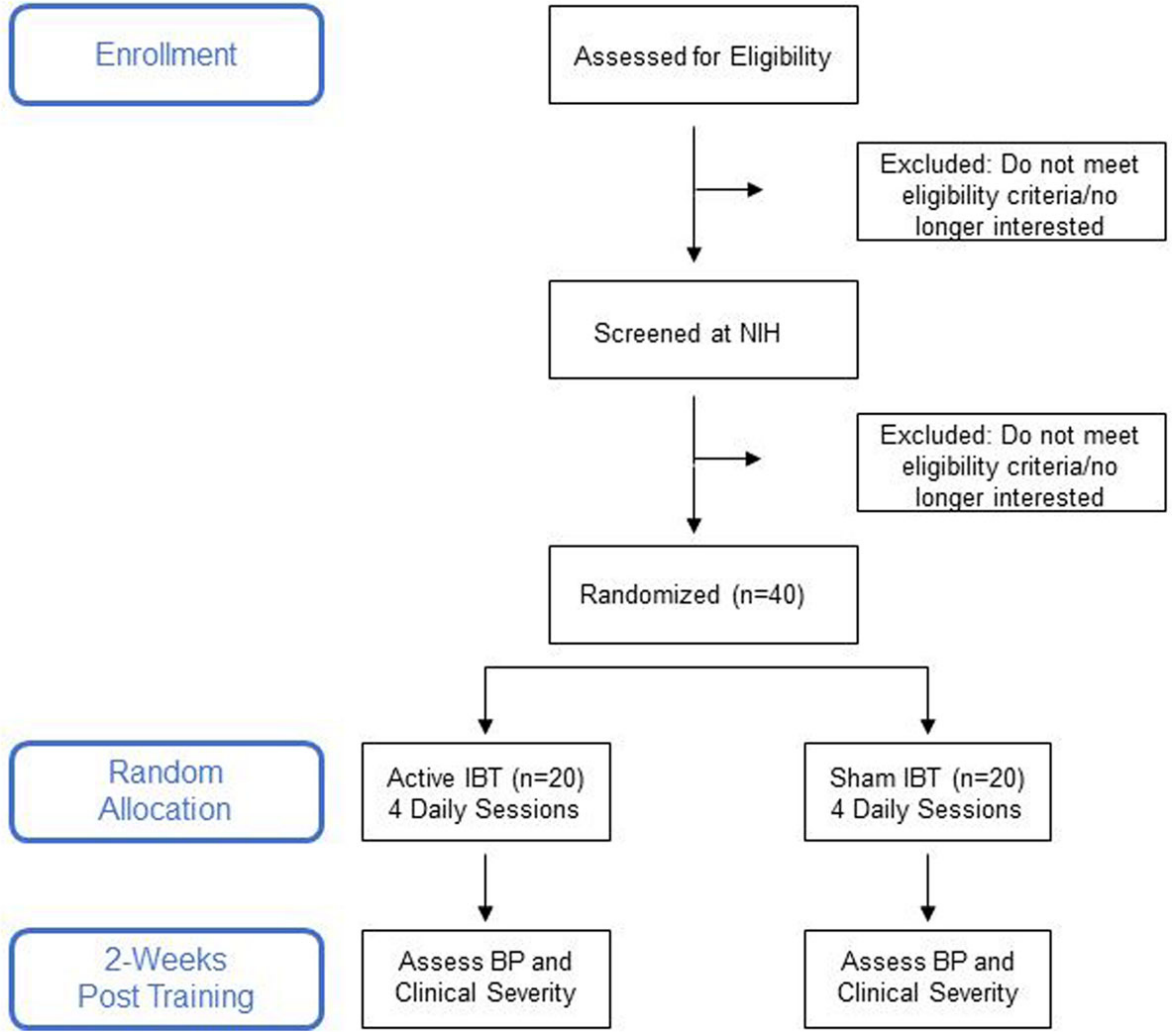
Flow diagram of the Interpretation Bias Task randomized controlled trial (IBT RCT)

The study described in this protocol performs a double-blind RCT to test the efficacy of IBT as an augmentation to standard of clinical care (i.e., psychotropic medications and/or psychotherapy) for DMDD (see Fig. 1). Forty youth with current DMDD will be randomized (1:1) to receive four IBT sessions (Active vs. Sham, in a double-blind design) over 4 days followed by weekly mood ratings and BP assessments for 2 weeks. Primary outcome measures in this trial assess changes in irritability using the Clinician Affective Reactivity Index (Clinician ARI) and Clinical Global Impressions-Improvement for DMDD (CGI-I; [25]), as well as parent- and child-report of irritability (child- and parent-rated ARI; [26]). Additional file 1 provides the SPIRIT checklist for this trial.

### Recruitment

Patients will be recruited nationwide through Institutional Review Board (IRB)-approved postcards, mailings, announcements in newsletters and bulletin boards or centers, and contacts with support groups and approved websites. Information leaflets may be posted electronically on websites such as National Institutes of Health (NIH) or National Institute of Mental Health (NIMH) websites, advocacy support group websites (e.g., National Alliance on Mental Illness (NAMI), Children and Adults with Attention Deficit/Hyperactivity Disorder), publications’ websites (e.g., *Washington Parent*). Researchers (MAB) also give presentations to the public and mental health professionals regarding our research and recruitment efforts for studies.

Informed consent and assent will be obtained from parents and youth, respectively, before any trial-specific procedures are performed. All participants will be advised that research is entirely voluntary, that they may withdraw participation at any time. Families will be paid for participation.

At screening and during enrollment of the IBT RCT, participants will be informed of, and agree to, maintain their current mental health treatment regimen 2 weeks prior to the start of the IBT RCT and through the second follow-up visit.

### Participants

#### Inclusion criteria

Participants must be between the ages of 8–17 years and currently meet full DSM-5 diagnostic criteria for DMDD as assessed by a structured clinical interview (Kiddie-Schedule for Affective Disorders and Schizophrenia Present and Lifetime Version; K-SADS-PL; [27]), with an additional supplement to assess the presence of DMDD [28]. Additionally, based on record review and interviews with child and parent, the research team must agree that the child’s response to their current treatment is no more than minimal as operationalized by a DMDD CGI-S (1-month version) score of 3 or more. Patients must be fluent in English; both parents and youth must provide written informed consent and assent, respectively.

#### Exclusion criteria

Participants will be excluded if the irritability symptoms are due to the direct physiological effects of a drug, or to a general medical or neurological condition. Additionally, participants will not be eligible if they meet DSM 5-criteria, as assessed by the K-SADS-PL (Kaufman et al., 1997), for schizophrenia, schizophreniform disorder, schizoaffective illness, autism spectrum disorder, posttraumatic stress disorder, current major depressive disorder, or if they meet criteria for alcohol or substance abuse 3 months prior to enrollment. Participants will be excluded from the study if they exhibit cardinal bipolar symptoms or have an IQ below 70 as assessed by the Wechsler Abbreviated Scale of Intelligence (WASI; [29]). Patients must not have planned changes in outpatient psychiatric treatment regimen, which can include psychotropic medications and/or psychotherapeutic interventions, 2 weeks prior to enrollment and throughout the 3 weeks of the RCT training and post-training follow-up assessments.

### Intervention

**Fig. 2:**
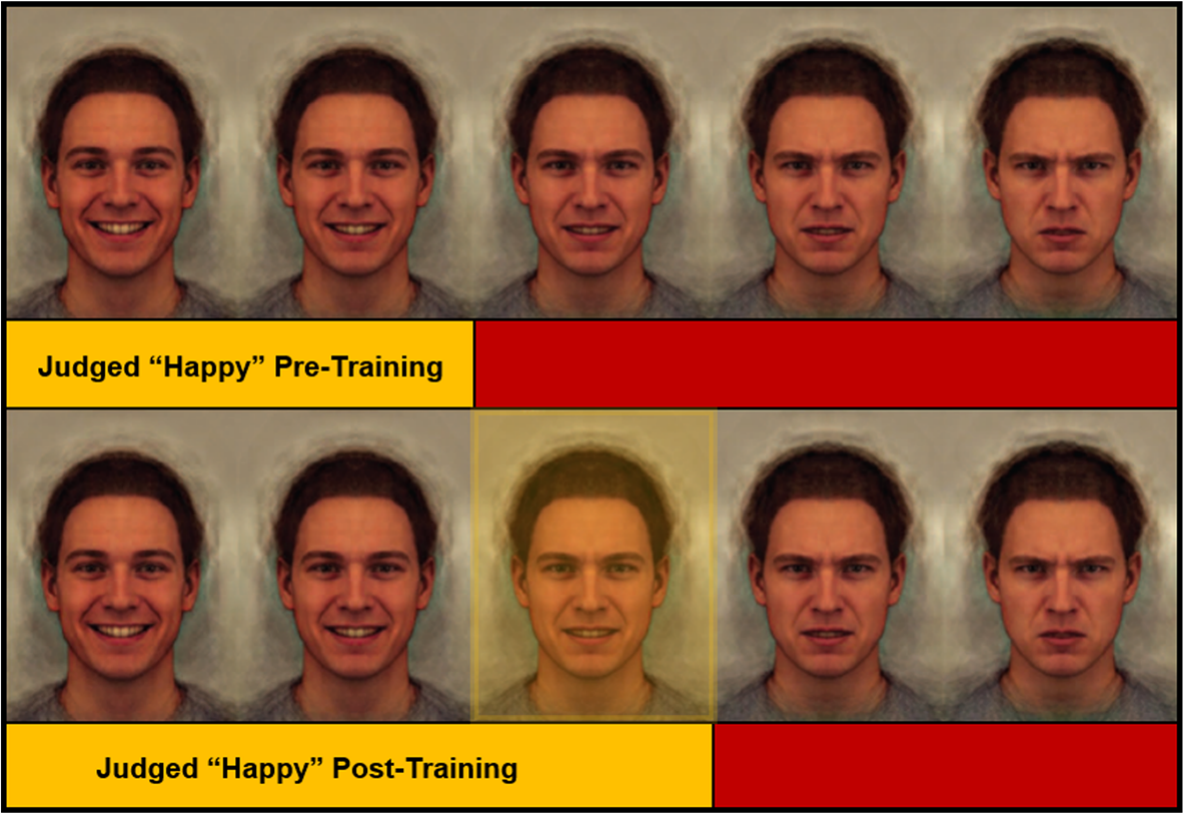
Training is designed to shift pre-training balance point, thereby reducing an angry interpretation bias. Adapted with permission from the authors [22]

The IBT task assesses and trains happy-angry interpretations of face emotions. There are two versions of the task, an assessment version (Assessment Task) and a training version (Training Task, either Active or Sham). The task stimuli for both tasks consist of the same set of 15 face-emotion pictures, or “face morphs” (see Fig. 2 for example stimuli [30]). Face morphs were created from two prototypical happy and angry composite images of 20 individual male faces from the Karolinska Directed Emotional Faces [24, 30]. These happy and angry prototypical images were used as endpoints to generate a continuum of 15 images from unambiguously happy to unambiguously angry.

#### Assessment Task

In the Assessment Task, morphs are presented three times in random order for a total of 45 trials. Each trial consists of a fixation cross (1500–2500 ms), image presentation (150 ms), visual noise mask (250 ms), and a screen (no time limit) where a person is required to make a forced-choice response of “happy” or “angry” via keyboard button press. The Assessment Task takes approximately 2.5 min. The Assessment Task measures the participant’s BP, i.e., the morph at which the participant shifts their judgment from predominantly happy to predominately angry, with the continuum of morphs coded on an integer scale from the happiest endpoint = 1 to the angriest endpoint = 15. The BP can be estimated in real time during a training session as the proportion of happy responses to total responses multiplied by 15.

#### Training Task

The Assessment Task is administered before and after each Training Task to measure BP pre and post each training session. In both the Active and the Sham training version of the task, the stimuli and timing are the same as the Assessment Task. The difference is that feedback is provided to the participant on every trial after their response. Feedback consists of a message saying “Right!/Wrong!” Consistent with previous IBT trials (e.g., [24]), in the Sham training, feedback is based on the participant’s original BP, as measured using the Assessment Task before training (i.e., the feedback is consistent with the participant’s pre-training BP). In the Active training, feedback is based on a modified version of the participant’s pre-training BP. Specifically, the Active training is designed to shift the participant’s BP so that two ambiguous morphs that were rated angry at pre training will be rated as happy post training. The feedback is based on the participant’s pre-training BP; hence two ambiguous images in the center of the morph continuum that the participant judged as angry pre training will instead be considered happy for the purposes of feedback. In the Training Task, each morph will be presented twice in random order to create a training block. There are six training blocks of 30 trials each summing to a total of 180 training trials.

#### Outcome measures

In addition to primary outcome measures assessing changes in irritability (ARI and CGI-I), this trial includes several secondary outcome measures. Secondary outcome measures include clinician ratings of depression, (Children’s Depression Rating Scale, CDRS; [31]), anxiety (Pediatric Anxiety Rating Scale, PARS; [32]), and impairment (Clinical Global Impressions-Severity for DMDD, CGI-S; [25]; Children’s Global Assessment Scale, CGAS; [33]). Additionally, parent-report measures of anxiety (Screen for Childhood Anxiety Related Emotional Disorders-parent, SCARED; [34]) and aggression (Modified Overt Aggression Scale, MOAS; [35]) and child self-report measures of depression (Children’s Depression Inventory, CDI; [36]), anxiety (Screen for Childhood Anxiety Related Emotional Disorders-child, SCARED; [34]), anger (State-Trait Anger Expression Inventory-2 Child and Adolescent, STAXI-2 C/A; [37]). We will also collect information on social status (Kids in My Class Questionnaire, [38]; MacArthur Scale of Subjective Social Status; [39]) and intent attributions (Hostile Attribution Bias [HAB]) using a hypothetical-situations instrument [40].

### Study procedure

**Fig. 3:**
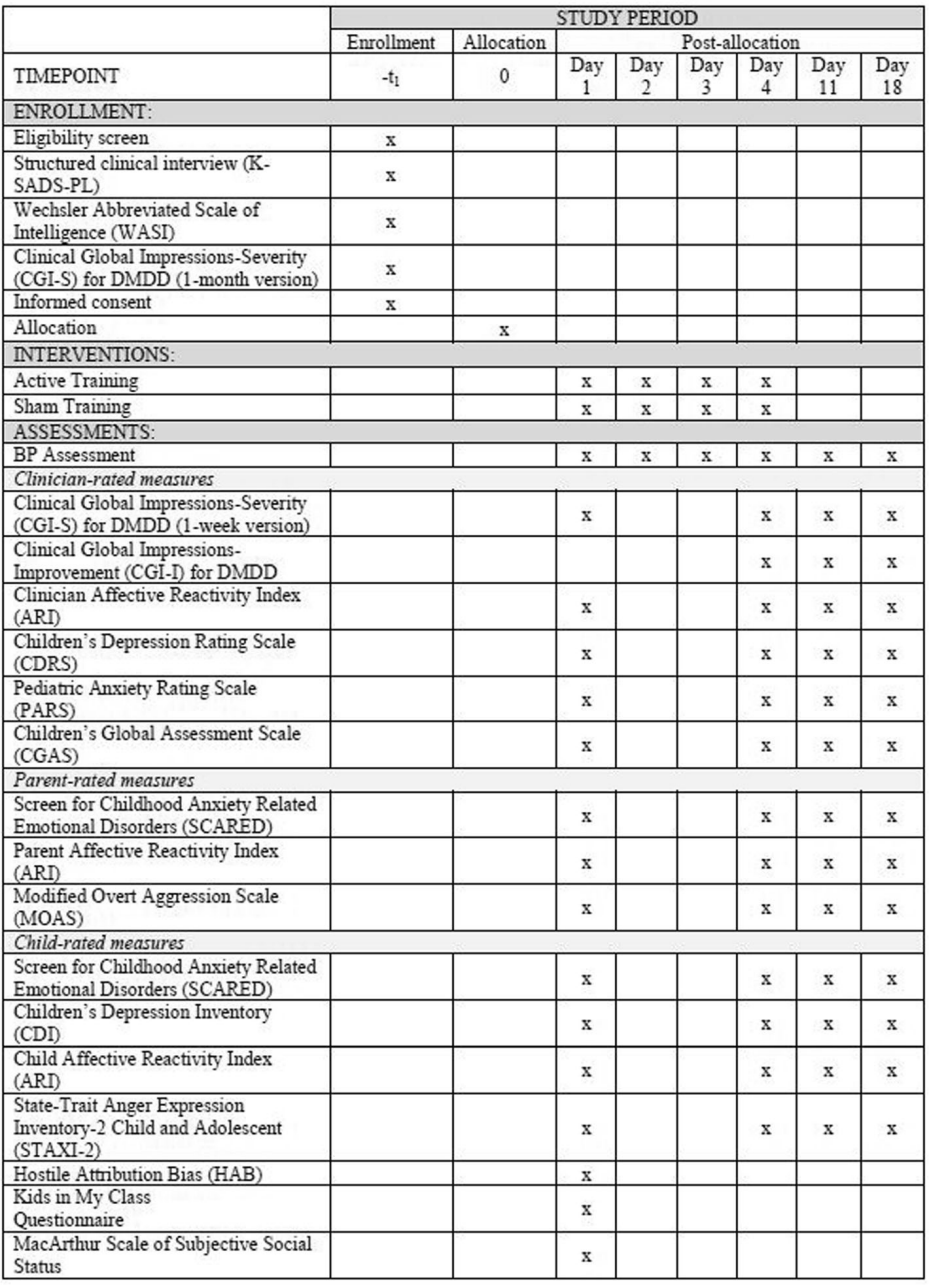
Schedule of enrollment, interventions, and assessments

Figure 1 provides a flow diagram of the study design, Fig. 3 provides a detailed schedule of enrollment, interventions and assessments. Participants will first complete a pre-training clinical screening before being enrolled in the protocol and randomized. Participants will be allocated to receive four IBT sessions (Active vs. Sham training in a double-blind design) over 4 days. Participants will complete a series of clinician- and self-report ratings pre and post training days followed by weekly clinical ratings and BP assessments for two additional weeks post training. The endpoint for primary outcome measures will be the DMDD CGI-I and ARI on the first follow-up visit (i.e., day 11). Figure 3 provides an overview of clinician-, parent-, and child self-report measures administered at the different time points. Participants’ intervention allocation for the entire cohort will only be unblinded upon completion of the IBT study.

### Pre-training screening visit

Participants and their parents will visit the NIH for a pre-training clinical assessment to determine eligibility to enroll in the IBT RCT. A trained masters- or doctoral-level clinician will conduct interviews with the participant and their parent including a current diagnostic interview (i.e., K-SADS-PL) and a clinical rating on the DMDD CGI-S.

### Training visits (days 1–4)

Custom IBT software, written in Tcl (*www.tcl.tk*) (see also [19]), will be packaged with stimuli for Mac and PC computers. During visit day 1, participants will complete the first session of IBT using either their own laptop computer or a NIH computer. All IBT data will be quality checked post training. Stimulus presentation timings will be inspected for each training session to ensure that timing of face displays is precise on non-NIH equipment. During this visit, participants and their parents are interviewed by clinicians for clinical ratings for irritability (cARI), depression CDRS), anxiety (PARS), overall functional impairment (CGAS), and severity of irritability and outbursts (CGI-S for DMDD, 1-week version). Participants and parents will also complete self-report forms. On day 2 and day 3, participants will complete one session of IBT, both sessions will be completed at home. The final training session on day 4 will again be onsite. On day 4, the participant and their parent will again be interviewed by clinicians for clinical ratings for irritability (cARI), depression (CDRS), anxiety (PARS), overall functional impairment (i.e., CGAS), severity of irritability and outbursts (CGI-S for DMDD), and improvement (CGI-I for DMDD). The CGI-I interval will cover the time from day 1 through day 4, and the rating will be relative to the day-1 CGI-S. During this visit, participants and parents will again complete all self-report forms. To allow for reasonable flexibility, during days 1–4, participants may reschedule up to two training sessions by extending the training period to day 5 or 6 to complete four once-daily sessions within a 6-day window. The participant completes rescheduled training sessions at home and self- and parent-report measures will be completed online. Clinical ratings may be done by phone.

### Follow-up visits

#### 1-week post training (day 11)

This follow-up visit determines whether training effects persist for 1 week. Participants will begin this visit with an onsite Assessment Task to measure BP. Next, the participant and their parents will be interviewed for clinical ratings. The CGI-I interval is the past week, and the rating is relative to the day 1 CGI-S. During this visit, participants and parents will complete the same self-report measures.

#### 2-weeks post training (day 18)

This follow-up is completed from home and determines whether training effects persist for 2 weeks. Participants will begin this session with an Assessment Task to measure BP. Next, the participant and their parent will be interviewed for clinical ratings. The CGI-I interval is the past week, and the rating is relative to the day-1 CGI-S. Participants and parents will complete self-report measures online.

### Allocation

Intervention allocation sequence will be created via a computer-based random number generator. Randomization will be performed in blocks of 10 participants with a 1:1 ratio within blocks. Study personnel not involved in other aspects of the study will perform randomization. The document with the allocation sequence will be unavailable to those that enroll or otherwise interact with study participants. Training tasks will be prepared by study personal not otherwise involved in the study and IBT training tasks (Active vs. Sham) will be formatted identically. Trial participants, outcome assessors, data analysts, and research assistants interacting with families will be blind to participants’ intervention allocation. The blind is not broken for the entire cohort until completion of the IBT RCT.

### Statistical analyses

#### Analysis of data/study outcomes

Primary outcome measures are ARI and CGI-I. We will analyze both primary outcomes measures as a function of training group (Active vs. Sham) and time point (pre training and 1 week following training (day 11)). All data will be analyzed in accordance with the intention-to-treat principle. Following Stoddard et al. [19] and Pollak and Kistler [41], we will fit a logistic curve to the binary happy-angry judgment data to derive the BP. We will examine associations among BP, clinical ratings, and treatment condition. We expect any symptom changes in the Active training group to be associated with shifts in BP.

#### Power analysis

The open pilot trial [19] found a large effect of IBT on outcome (*d* = 1.2 for CGI-I). Because studies with similar computer-based approaches (see [42] for a meta-analysis) have found more moderate effect sizes in RCTs, we expect an effect size of *d* = 0.8 to *d* = 1.0. Power analyses suggest that 20 per group will be sufficient to detect differences at alpha = 0.05, beta = 0.20. Dropouts will be replaced for an *n* = 40 of participants who completed the trial.

### Ethics

Study procedures were approved by the National Institute of Mental Health (NIMH) Institutional Review Board (IRB). Risks associated with participation in this RCT are considered minimal. Adverse experiences include mild psychological distress and discomfort when responding to interview questions or completing questionnaires, or boredom during the IBT task. The principal and associate investigators will monitor patients closely throughout their participation in the RCT. After completing the RCT, all participants will be offered four Open Active IBT sessions, delivered on the same schedule as in the RCT and using the same primary outcome measures. For the Open Active IBT, while the principal and associate investigators will continue to monitor the patient, community providers are overseeing the patients’ care.

Participants will be informed of their RCT intervention allocation upon completion of the trial.

An independent safety monitor will surveil data and safety. Audits of this treatment protocol will be conducted on a regular basis.

## Discussion

This study is the first RCT of IBT in youth with DMDD. Chronic irritability, such as observed in DMDD, is severe and impairing for affected youth and their families, yet available treatment options are limited (Brotman et al., 2017). The inclusion of DMDD in the DSM-5 demonstrates the need to develop evidence-based treatments to target severe, pathological irritability. The current protocol describes the first double-blind RCT aimed to test the efficacy of IBT in reducing irritability in youth with DMDD. Forty youth are randomized to receive either four Active IBT or Sham IBT sessions in a double-blind design. Active IBT is designed to shift participants’ pre-training BP along a face-morph continuum towards making increasingly happy judgments on ambiguous face morphs. Sham training does not change the participants’ pre-training BP. BP is measured 2 weeks following treatment. Primary outcome measures assess changes in irritability pre to post training and 2 weeks after training using the clinician ARI and CGI-I for DMDD, as well as parent- and child-reports of irritability using the ARI. Secondary outcome measures include clinician ratings of depression, anxiety, and impairment. Parent- and child self-report measures of depression, anxiety, anger, social status, and aggression are also collected.

In this RCT, IBT is an augmentation to stable community treatment (pharmacological and/or psychosocial). Developing non-pharmacological treatment options for youth suffering from severe, chronic irritability is particularly important to minimize psychotropic medication exposure with profound side effects on the developing brain. Additionally, computer-based interventions have the potential to help bridge the treatment gap in psychological care as an easily accessible, cost-effective tool.

### Trial status

The protocol (15-M-0182, Psychological Treatments for Youth with Severe Irritability) received final IRB approval in August 2015, after which trial recruitment commenced. Recruitment is expected to be completed by January 2019.

### Limitations

This study has several limitations. First, the sample size is modest to investigate potentially small therapeutic effects. Second, follow-up ratings will be conducted over 2 weeks following the intervention. Therefore, the study is limited in its ability to assess longer- term or delayed effects on symptoms. Third, we will not test near or far transfer of learning; face interpretations are trained and tested on the same face set. Should we find significant symptom reductions in the training group, examining whether training effects (i.e., changes in BP) generalize to new face stimuli would be a crucial next step to provide evidence for the mechanism by which active IBT may be efficacious. Lastly, we will only assess IBT as an add-on to stable community treatment. Participants enrolled in the trial will be concurrently taking a variety of different medications, including psychotropic medications. However, as discussed, we do require medications to remain stable throughout the trial.

## Additional file

Additional file 1: SPIRIT 2013 Checklist: Study protocol for a double-blind, randomized, placebo-controlled trial of a computer-based Interpretation Bias Training for youth with severe irritability. (DOC 123 kb)

## Abbreviations

ARI: Affective Reactivity Index
BP: Balance point
CDI: Children’s Depression Inventory
CGAS: Children’s Global Assessment Scale
CGI-I: Clinical Global Impressions-Improvement for DMDD
CGI-S: Clinical Global Impressions Severity for DMDD
DMDD: Disruptive mood dysregulation disorder
DSM 5: *Diagnostic and Statistical Manual of Mental Disorders 5*
HAB: Hostile Attribution Bias
IBT: Interpretation Bias Training
IRB: Institutional Review Board
K-SADS: Kiddie-Schedule for Affective Disorders and Schizophrenia
MOAS: Modified Overt Aggression Scale
NIH: National Institutes of Health
NIMH: National Institute of Mental Health
PARS: Pediatric Anxiety Rating Scale
RCT: Randomized controlled trial
SCARED: Screen for Childhood Anxiety Related Emotional Disorders
STAXI-2 C/A: State-Trait Anger Expression Inventory-2 Child and Adolescent
